# Clinical Focus on Prosodic, Discursive and Pragmatic Treatment for Right Hemisphere Damaged Adults: What's Right?

**DOI:** 10.1155/2011/131820

**Published:** 2011-02-16

**Authors:** Perrine Ferré, Bernadette Ska, Camille Lajoie, Amélie Bleau, Yves Joanette

**Affiliations:** ^1^Centre de Recherche de l'Institut Universitaire de Gériatrie de Montréal (CRIUGM), Québec, Canada H3W 1W5; ^2^Hôpital de Réadaptation Villa Medica, Montréal, Québec, Canada; ^3^Université de Montréal, Québec, Canada H3T 1J7

## Abstract

Researchers and clinicians acknowledge today that the contribution of both cerebral hemispheres is necessary to a full and adequate verbal communication. Indeed, it is estimated that at least 50% of right brain damaged individuals display impairments of prosodic, discourse, pragmatics and/or lexical semantics dimensions of communication. Since the 1990's, researchers have focused on the description and the assessment of these impairments and it is only recently that authors have shown interest in planning specific intervention approaches. However, therapists in rehabilitation settings still have very few available tools. This review of recent literature demonstrates that, even though theoretical knowledge needs further methodological investigation, intervention guidelines can be identified to target right hemisphere damage communication impairments in clinical practice. These principles can be incorporated by speech and language pathologists, in a structured intervention framework, aiming at fully addressing prosodic, discursive and pragmatic components of communication.

## 1. Introduction

Communication disorders following a right hemisphere stroke are nowadays well documented in literature. To date, research has mainly been devoted to the description of communication disorders in adults with right hemisphere damage (RHD) with the goal of better understanding their clinical and neuroanatomical correlates. The availability of specific assessment tools has contributed to a proper understanding of the peculiar semiology of these deficits. The impetus of neuroimaging techniques also provided advanced knowledge on the specific contribution of the right hemisphere to language and communication. 

More recently, researchers have focused on intervention with RHD individuals. For instance, nonverbal components have been addressed extensively in the literature (i.e., visual neglect) but there is still a critical lack of reports on rehabilitation for three of the most important components of communication affected after a right hemispheric stroke: discourse, semantics, and prosody. 

However, clinical expertise is available and theoretical guidelines can be inferred from previous studies. This primary knowledge already constitutes a useful body of evidence in clinical settings.

Thus, we propose a recent review of the literature and clinical descriptions, aiming at providing hints for clinicians to elaborate an adapted intervention for RHD individuals. 

## 2. State of the Art

### 2.1. Incidence

It has been estimated that between 50% [1] and 78% [2] of RHD individuals may exhibit difficulties in one or more communication components, leading to inadequate social interactions. It is well known that clinical manifestations evolve over time after a brain lesion. This could explain, at least partially, the discrepancies reported in the percentage of RHD patients effectively presenting with communication disorders. Thus, the time postonset at which participants are recruited is a key variable to take into consideration. So far, no longitudinal study has been undertaken with RHD adults regarding the progression of communication deficits over time. The sensitivity of assessment tools used to detect communication deficits might also play a role in the inconsistency of the data, as it will be discussed later. 

### 2.2. Characterization of the Deficits

Right hemisphere lesions might affect four different components of verbal communication: prosody, discourse, semantics, and pragmatics. Each one of these aspects has been thoroughly described in scientific literature for the past 20 years (for a complete review, see Myers [3] and Tompkins [4]). Nevertheless, patients can present one or more communication impairments and thus be gathered according to their specific profile, as it has been done with left hemisphere aphasia sub-types (e.g., Broca's, Wernicke's, Anomic aphasia). Recently, four distinct clinical profiles have been described in a population of 112 RHD adults from three different countries [1]. In all clusters but one, RHD adults are impaired in their conversational discourse. The first cluster is mainly characterized by prosodic impairments. Cluster two exclusively displays conversational discourse disorder. The third cluster presents low-to-moderate impairments in narrative (and not conversational) discourse, semantics, and emotional prosody. Cluster four, interestingly, shows extensive and more severe impairments in all components. Variables such as age, education, time postonset, type, or site of the lesion do not seem to play a significant role in the communicative behavior of the subgroups. However, at an individual level, a recent preliminary study [5] indicates that participants with hemorrhagic strokes tend to show more severe deficits in conversational discourse when compared with ischemic stroke participants. This observation is in accordance with the expected clinical consequences of a subcortical lesion, as it is more frequently the case when the nature of the stroke is hemorrhagic [6].

It has been suggested that the relation with other cognitive disorders, including disexecutive syndrome (mental flexibility, inhibition, shared attention mechanisms) should be further explored [3]. Several authors addressed this issue by studying each component of communication independently. These conclusions will be discussed further in the paper through the description of deficits. Preliminary results in a comprehensive analysis of communication and executive components indicates that executive processing can have a direct or indirect impact on the pragmatic, discourse, and semantic components of communication [7]. For example, indirect speech acts require simultaneous processing of various types of information about context and semantic and might therefore highly depend on the integrity of the entire cognitive system. On the other hand, the ability of prosody seems to depend on the lesion's localization and on its impact on the connectivity network in the right hemisphere. Lajoie concludes that a cognitive-executive disorder will increase the degree of severity of a related communication disorder rather than causing it. Other cognitive abilities have been scrutinized and held responsible for some or all of the communication deficits presented by brain damaged patients. In reference to the possible link between the Theory of Mind (ToM) and language deficits (as claimed by Champagne and Joanette [8]) many discrepancies appear in the literature. Initially, ToM relied on the premise that successful communication depends on the ability to infer the mental state of the speaker. Tompkins et al. [9] state that inadequate stimulus control and imprecise measure of causal inference might account for deficits described in previous studies. Though limited by the use of only one type of ToM task (“cognitive ToM”, in opposition with “social, emotional ToM”), Tompkins emphasizes the false interpretations possibly linked to the use of stimuli that were too easy to reveal communication impairment profile in RHD individuals. Accordingly, Griffin et al. [10] stressed the fact that RHD adults have specific difficulties to attribute intentional states that involve second-order attributions. In addition to accentuating the need for further exploration in many aspects of RHD deficits, these studies highlight the need for adequate control of assessment tasks and stimuli selection that rely on up-to-date theoretical knowledge.

### 2.3. Assessment

Researchers using traditional aphasia batteries claimed that language disorders were not to be expected in RHD adults [11]. It has now been shown that tasks of high complexity will intensify the deficits observed in the performance of RHD individuals [12]. Moreover, the use of highly structured tasks will lead to great variability in RHD performance [13]. Therefore, clinicians should favor an assessment matching both structured and natural tasks' settings. As an integrative part of the rehabilitation process, evaluation is briefly described here to guide clinicians in their choice of the assessment tools. 

Specific formal structured assessment tools are available in various languages. In English four batteries have been published: *The Ross Information Processing Assessment* [14], the Mini Inventory of Right Brain Injury [15], the *Right Hemisphere Language Battery* [16], and the *Rehabilitation Institute of Chicago Evaluation of Communication problems in right hemisphere dysfunction Revised* [17], in French, *La Gestion de l'Implicite* [18] that targets the indirect language; a test developed by Champagne et al. [19] assesses communication intentions; Chantraine et al. [20] have developed a task to target specifically the ToM; *Le Protocole Montréal d'Évaluation de la Communication* [21] that addresses all the aspects of communication. An English adaptation of the *MEC protocol* is under elaboration. The same protocol has also been produced in Spanish (*Protocolo MEC* [22]) and Portuguese (*Bateria MAC* [23]). Still clinicians should consider that all of these instruments have been published more than ten years ago and have psychometric and theoretical limitations.

To assess communication in a naturalistic way, conversation and communication grids constitute appropriate tools to address functional outcome measures, as underlined by Odell et al. [24]. Among other pragmatic tools, the *ASHA functional communication measure *[25], or *the functional assessment of communication in adults* [26] are well adapted to this population.

As reminded by Côté et al. [11], the evaluation should always consider the preonset personality and communication habits, the functional impact on personal and professional life of the communication deficits, as well as the sociocultural level. In that perspective, the dialogue with the proxies is a necessary step. This would enable a clearer perspective on the possible changes in the communication behavior and would offer an opportunity to inform and to involve the proxies in the therapy process. Moreover, a questionnaire presented to the patient himself will provide, when compared to proxies and therapists' opinion, a first look on the awareness of deficits, and an estimate of the client's request to set the intervention. This kind of tool might be used at different times of treatment to address the progress in terms of deficits' awareness, language performance, and client's satisfaction. Patient and proxies reported outcomes can reliably be objectivized with specific questionnaires. The BOSS [27] estimates the functioning and the well-being of stroke survivors with and without communication disorders. The CETI [28] assesses quality of everyday communication through a 16-point questionnaire addressed to the relatives. In the CAL [29], patient and proxies evaluate separately the amount and quality of everyday communication compared with prior to the stroke the stroke for 10 items. Some global assessment protocols also include questionnaires [21]. It has been noted that those tools are in some cases more sensitive to improvements in everyday-communication than standardized tests, especially regarding time-course evolution [30]. 

Therefore, the assessment step provides a better perspective at the difficulties encountered by the RHD individual, his family, and the clinician. Additionally, it is crucial to decide the usefulness and feasibility of a communication therapy as well as the goals and means of intervention.

### 2.4. Context of Intervention

In part because of inadequate assessment tools, RHD individuals have for a long time been excluded from the language therapeutic system. This population lacks systematic reference in SLP since the priority has been given to motor, visual and other cognitive disorders [11]. Clinicians still have demands for theoretical and practical expertise with this specific population. The same study also observed that therapies with RHD adults appear less rewarding for clinicians, considering the frequent associated cognitive disorders (i.e., anosognosia, impulsivity, frustration, and irritability). 

In 2005, the *functional independence measure* (in [24]: *FIM*; Center for Functional Assessment Research), although widely used in clinical settings by pluridisciplinary teams, has been proven to address inadequately the manifestations of cognitive and psychosocial disabilities that can result from RHD. The authors infer that this unexpected result also reflects the poor knowledge of the health care staff. In fact, it can be stated that although knowledge transfer has increased in recent years, clinicians still feel insufficiently prepared to treat communication disorders following RHD.

### 2.5. Treatment Efficacy

According to Cicerone et al. [31], there is substantial evidence to support cognitive rehabilitation for people with TBI. Significant evidence demonstrates that visuospatial rehabilitation is efficient for deficits associated with visual neglect after right hemisphere stroke. In fact, a great number of studies exists on neglect, even though neglect is not a reliable predictor of the global outcome for RHD adults [24]. 

As stated by members of the task force of cognitive rehabilitation [32], pragmatic-conversation therapy is based on a particularly limited number of studies. The scientific community particularly regrets that no evidence-based treatment is available to clinicians [33]. Nonetheless, review of literature since the year 2000 demonstrates that a fair number of reports on RHD rehabilitation exist. This literature offers valuable information based on single-subject designs or clinical and theoretical knowledge. Regardless of the differences in clinical profiles originating from a stroke or a traumatic brain injury (TBI), complementary information may also be collected from intervention studies with the TBI population. They benefit from a stronger scientific literature that demonstrates encouraging results regarding treatments of communication disorders. See Ylvisaker and colleagues [34] for a review. 

## 3. Communication Deficits and Treatment Studies

### 3.1. Prosody

Individuals with RHD might show monotonous speech [35, 36], a lack of facial expression [37] or atypical speech rate [35]. On the receptive side, some RHD adults exhibit difficulties to understand the emotion provided by the communication partner [35]. Disturbances are particularly observed at a syntactic level [38]. Theoretical and anatomical correlates are still under discussion. Some authors describe a specialization of the right hemisphere for the processing of emotional prosody and of the left hemisphere for the linguistic prosody [38]. Distinctive types of aprosodias have therefore been associated to specific lesion sites. Each profile has also been linked with impaired executive functions (e.g., inhibition, flexibility, and working memory) when semantics is not congruent with the prosody used [39]. On the other hand, several authors argue that the right hemisphere is responsible for both emotional and linguistic modalities [36, 40], emphasizing the interaction with the left hemisphere for the integration of linguistic elements of speech with prosodic cues. Another hypothesis claims that the linguistic and emotional prosody would be managed by subcortical structures, particularly the basal ganglia [35, 41]. This hypothesis implies that motor deficits account for the production of emotional prosody. Furthermore, Schirmer and Kotz [42] recently proposed a processing model of emotional prosody through several chronological steps occurring in various cortical and subcortical sites. In sum, prosodic processing, because of its multidependent position in language, relies on numerous structures and requires a series of complex cognitive operations.

**Intervention**

In the last decade, the literature on prosody treatment has proliferated. Leon et al. [43, 44] have studied two types of treatment for emotional prosody with five and more recently with fourteen patients. The first type is based on a motor speech theory for aprosodia and therefore suggests motor-imitative exercises of higher complexity (through a six step hierarchy of cues). The second type of treatment aims at restoring the knowledge between emotions and intonation production [45]. The study found that both types of treatment had some positive impact.

In a preliminary study, Guillet [46] elaborated an intervention following three guidelines: awareness, hierarchy, and consideration of cognitive impairment. After a preliminary phase of discrimination between emotional and linguistic sentences, the receptive phase took place. Participants were expected to identify words and sentences, with and without respect of the syntactic borders. In the productive step, patients were then expected to repeat, read, and create dialogues using the proper intonation. PACE situation (cited in [47]) and phone conversation were ultimately practiced. The two RHD patients showed significant gains in both expressive and receptive prosody (linguistic and emotional) whereas the control participant did not. The proxies also noted a positive change in everyday life, suggesting a possible transfer.

Another recent exploratory case study [48] used the same treatment design but included a visual feedback (piano keys) for both receptive and expressive goals. Results tend to show a gain in all prosody modalities except in the comprehension of linguistic prosody.

In a more compensatory perspective, the patient can also bypass its prosodic deficits by relying on semantics in order to convey his emotions. Proxies should also be well informed of the neurological bases of prosodic impairment in order to avoid misunderstanding the psychological state of their relative [49].

### 3.2. Discourse

Most of our knowledge on discourse comes from studies of narrative discourse. Narrative discourse in RHD adults might be less informative, even if the number of words is similar or superior to controls. The content might be incoherent, tangential, and self-oriented [50]. Moreover, in a conversational discourse, some RHD individuals have difficulties sharing the responsibility to develop and maintain adequately the exchange with the speaker [51]. On the receptive side, they might have difficulties retelling a story as a whole [52]. Again, the complexity (e.g., inferences, absence of title, conversation in natural context, etc.) can highly influence performances [53]. 

Conversational discourse constitutes the most complex communicative situation, since it requires that each speaker uses all his linguistic skills in a given context. Therefore, conversational discourse especially relies on competences of the two hemispheres. The left side would be in charge of basic information (word recognition, syntactic processing), whereas the right hemisphere would be activated when processing higher level information (integration of parts as a coherent whole) [54]. Consequently, different authors argue that cognitive disorders would account for disturbed discourse skills [3, 4, 55, 56]. It is important to observe that discourse is inseparable from pragmatic abilities (as it will be discussed later).

**Intervention**

Regarding discourse treatment and RHD, the literature is sparse. Nevertheless, cues on discourse intervention have been recently proposed [57], targeting the increase of relevant information in RHD individuals. Several types of stimuli (e.g., a picture, a story told verbally or a video watched by both the patient and the clinician) are recommended to encourage discourse production. The main information has to be retold and the inferences discussed. Verbal production can be recorded in order to be listened and discussed subsequently. With the aim of increasing the level of difficulty, the stimuli can be put out of sight of the participant or be related to a less familiar subject.

In the absence of evidence, other cues based on theoretical hypothesis were suggested by Blake [33]. For instance, she suggests a treatment based on the acknowledged difficulties to use the context, by discussing alternate words or sentences' meaning according to a given context. If the clinician wishes a treatment based on presumed ToM deficits, he could choose inferences involving the integration of multiple cues. For instance, the client could be asked to identify the relationship between the speakers, their different points of view, or the meaning implied by the tone of their voice. In order to do so, scenarios can be created or debated in individual and group discussion sessions.

Treatment can also be managed by manipulating the difficulty level. Clinicians can control the number of cues or distractors, the length of the passages, the distance between the cues and the intended meaning or the number of people in the situation. None of those clinical ideas have been formally tested on RHD patients so far. Three recent preliminary case studies [48, 58, 59] have focused on goals such as maintaining the theme, sharing knowledge (in terms of quantity and accuracy) and turn taking following three guidelines [60]. The first step aimed at the arousal of deficit awareness. Participant had to identify the amount and accuracy of information according to the speaker, supported by a visual (pictograms of main information) or verbal feedback (questions on missing information). Therapists would then ask the patient to retell narratives he/she heard, to imagine himself/herself engaging in debates and controversy topics while decreasing verbal and visual feedback. Apart from the previous study from Klonoff, mentioned previously in former reviews [33], to our knowledge, no other formal study on discourse with RHD has been conducted.

### 3.3. Pragmatics

RHD individuals tend to show a preference for the literal meaning of expressions [61]. They sometimes have difficulties understanding unconventional indirect requests and new metaphors [8]. Additionally, RHD adults exhibit difficulties to govern verbal exchange since they take little account of their communicative partner (poor eye contact, incoherent or inappropriate comments, breach in turn taking, and insufficiently considered shared knowledge [20]). Finally, individuals with RHD injury, especially when reaching the frontal cortex, might also have trouble understanding humor and do not react physically to emotions (laughing or smiling) [62]. Here again, several authors have stressed the link between mental flexibility and understanding of metaphors [63], indirect speech acts [8], and inferences [64]. For all these aspects of communication, mental flexibility is needed to access figurative language. RHD adults could have difficulties disengaging from the literal meaning activated first or to revise their initial interpretation to make appropriate inferences.

As discussed earlier, pragmatic difficulties might also be linked to an inappropriate ToM [8], even though this aspect is controversial. Pragmatics, considered by some as a core component of communication, has been related to other executive, attention, or visuospatial disabilities [9].

**Intervention**

Literature devoted to TBI population provides some valuable hints on pragmatic intervention as well. Considering that the discoursive and pragmatics components are hardly dissociable one from another, treatments often address both. A review of 19 studies [65] focusing on social communication for individuals with acquired brain injury (almost exclusively TBI) demonstrates that the combination of different approaches increased the effect size in many clinical populations. Intervention methods with TBI traditionally employ applied behavior analysis (ABA) and more recently positive behavior supports (PBS). Both are practiced in individual or group therapies.

The ABA focuses on specific behaviors taught explicitly in a sequential manner, whereas the PBS focuses on lifestyle change satisfaction through internal control of behavior in natural settings. In practice, therapies use mostly the same features (e.g., modeling, role-playing, visual and verbal feedback, self-monitoring, behavioral rehearsal, and social reinforcement). However, it is worth observing that the success of traditional social skills training assumes, among other things, that the patient lacks explicit knowledge of relevant social rules, that he is motivated to change his social behavior, and that he possesses the capacity to transfer to various real-world situations skills acquired in a training setting [66]. Those assumptions are, most of the time, in opposition to RHD or TBI adults' behavior, especially in the case of anterior lesions.

These studies show us that decontextualized social skills training produces minimal effects on real-world behavior. In terms of maintenance in acquired abilities over time, two recent studies [67, 68], using the cited features, reported improvements in a six-month followup with TBI adults. Even though the targeted goals were not exclusively pragmatic-specific and that the individual characteristics among the groups were heterogeneous, the conclusion is that participants showed improvement in subjective measures of social communication skills posttreatment and at followup. One can only suppose that the RHD population, that might be partly similar in terms of behavior, might benefit from behavioral therapies based on methods using natural contexts. Maintenance of performance and satisfaction should however be evaluated for this specific population.

To specifically target the pragmatic field, some treatment studies [48, 58, 59] used barrier tasks (reconstitution of abstract figures using geometrical shapes) and procedure tasks (how to make or do) as well as role playing in several everyday situations. The goal was that the patient would adapt constantly the quantity and accuracy of information to the communication partner.

Gains, although inconsistent, were observed in the three studies relative to shared knowledge, understanding of communication intentions, maintenance of the theme, and social uses. Nevertheless, these clinical and exploratory studies employed weak methodologies that would be worthwhile reproducing with stronger designs.

A similar study [69] addressed exclusively the understanding of indirect speech acts. The tasks suggested were very functional, as therapists had to insert indirect commentaries during a common activity (such as looking for a journal article). The activities and related indirect commentaries are suggested by the author. The feedback was controlled and progressively decreased through therapies. The material was tested by one SLP with only one patient and no pre-post therapy evaluation. Nonetheless notable gains were reported, especially regarding the awareness of deficits.

A pilot study used a process-oriented (versus task-oriented) training [70]. The training is based on a simple model to represent the mental states of others through the use of thought bubbles [70, 71]. The patient is asked to determine the thoughts and to predict behaviors. The program begins with first-order beliefs and progresses to include second-order beliefs. The two patients seemed to show clear evidence of response to the training, but results should be validated.

## 4. Deduction of Intervention Guidelines for Clinicians

Considering evidence-based treatments for RHD adults, Blake [33] underlines that treatment can be either theoretically motivated or based on deficits. Looking at accounts of normal right hemisphere function, the current knowledge doesn't seem sufficient to totally rely on the latter option. On the other hand, treatment can be based on deficits rather than etiology. Considering the poor evidence in the field of practice for RHD communication treatment, clinicians can select treatments from other populations, especially adults with TBI. Indeed, Coelho et al. [72] claim that comparable discourse disorders may be observed secondary to focal and diffuse brain pathology. Clinicians also corroborate similarities between the two populations. However, interpretations should be made carefully [32] as we do not have tangential evidence so far to state whether those two populations are comparable. 

Even though no precise conceptual framework or evidence-based practice is yet available, some clinical guidelines are useful for clinicians willing to elaborate an appropriate intervention with their RHD clients. Also, valuable knowledge can be deduced from previous scientific studies cited earlier. Indeed, even if the literature reports heterogeneous results, some common features seem to emerge.

The actual trend states that, after the assessment phase, it is important to set an intervention that will answer the communicational and functional needs of the individual. The person and his proxies should therefore choose the intervention goals together with the therapist in order to set a collaborative base. As underlined by Tompkins [4], the goals should target the most prejudicial deficits in everyday communication activities. The proxies have to be involved all along the therapy to obtain a better generalization to different contexts. 

In order to motivate both the patient and his proxies -and considering that the theoretical framework remains unclear-therapy would preferably be task-oriented [3]. This approach aims at improving a specific function of everyday life using facilitatory and compensatory techniques. The benefits are expected to be immediate in everyday life, but since the therapy is very individualized, generalization to the whole population is hardly attainable. On the other hand, the choice of a* process-oriented* approach would imply that all cognitive processes underlying the language abilities are well acknowledged, which is not the case at this time with RHD adults. 

Even though there is great variability of profiles among RHD adults, three guidelines seem relevant to consider their common behavioral features. These guidelines are to be alluded to under different terms and separately through most of the studies mentioned above. Pauzé [60] explored how they could be integrated in one unique clinical framework.

1. Raise the awareness of the deficits. As it has been done with behavioral treatments for TBI adults, initial consciousness is necessary to help the individual perceive his acquired disorders as well as the benefits he could gain from therapy. RHD individuals can, to that end, listen to or see recorded sequences illustrating communication difficulties, first casting other persons and then with themselves in interaction. Clinicians can also suggest real-life examples and analogies, through a pictographic symbol illustrating the maladapted situation of communication (e.g., a highway illustrating the main theme, and exit roads suggesting the diverging commentaries). Symbols can be used during therapy and progressively replaced by verbal signals or gestures to diminish feedback and to abide by the second guideline.
2. Organize into a hierarchy. Almost all studies agree that activities as well as stimuli should be presented in progressive difficulty. Task difficulty can be manipulated through the type-modality (discrimination, identification, production), the answer-modality (multiple choice, free) and the procedure employed with time (speech rate, time allowed to complete the task, etc.). As it comes to stimuli, it is possible to vary the level of difficulty by modifying the perceptibility of stimuli (visual versus verbal presentation, unimodal versus multimodal, font, size, background, etc.) by changing their internal characteristics (frequency, imageability, organization in space, etc.) or by multiplying the contexts (structured to various complex natural settings).
3. Take into account the basic cognitive impairments (e.g., memory, attention, mental flexibility). Even though the literature still has to shed light on the links binding communication and other cognitive disorders, there is no doubt that the whole cognitive system is potentially affected following right hemisphere damage. Initially, this can be done by offering a facilitative context appropriate for each type of deficit which can be withdrawn in order to adopt a more realistic and complex context of communication. Each facilitator should be adapted to the specific disorder (e.g., by controlling the presentation of the stimuli in the visual space for hemineglect patients).

Altogether, recent the literature and research offer a primary framework for intervention with RHD populations, mainly advocating these three guidelines. Even though the conceptual framework is still unclear, clinicians have the necessary hints at hand to elaborate appropriate therapies. Despite the growing body of evidence, forthcoming challenges are to be expected.

## 5. Orientation for Forthcoming Studies

The outstanding challenge for researchers will be to systematically conduct studies with stronger methodology, may they be single-case or group studies. 

### 5.1. Experimental Design

In recent years, the literature has demonstrated the urgent need for formal pre-post assessment, clear and standard therapy frameworks as well as control groups. Those conditions are preliminary to any conclusive assumption on evidence-based rehabilitation after right hemisphere stroke. Considering the actual state of the art, single case studies are so far considered closer to clinical reality. Group studies should preferably wait for the establishment of clearer subgroups among the RHD population [11]. Also, considering the poor knowledge in terms of treatment efficacy with that population, single case studies will contribute to the identification of effective treatment methods [61, 72]. Randomized clinical trials should carefully consider the use of control groups. In order to draw conclusions based on the comparison of results in the experimental and in the control group, the choice of the tasks and activities given to the control group should be ethically and scientifically determined. It is not acceptable to propose no activity to the control group, knowing that any given activity could potentially benefit the experimental group. Taking into account that no “reference” treatment is available with RHD adults and communication disorders, pragmatic randomized trials could be preferred, as they test effectiveness in everyday practice under flexible conditions. The use of parallel groups would also allow comparison of different treatment methods in two groups. Furthermore, the consideration of cognitive deficits is necessary during the assessment phase, in order to examine in depth the relation between language and other cognitive components. As it has been done with left-hemisphere damaged individuals, an account of neural network using neuroimagery pre- and posttherapy would surely bring new light into the recovery and compensatory networks at stake after an RHD. 

### 5.2. Population

As suggested by Blake [33], it would be wise to compare communication deficits caused by different etiologies (focal versus diffuse damage) in order to establish whether similar interventions could be designed for both populations. 

### 5.3. Generalization

Future studies should seek generalization among tasks, individuals, and conversational contexts. So far, studies report very variable generalization. For instance, in a study on prosody by Leon et al. [43, 44] results might be poor to untreated emotions but generalized to untreated sentences. Activities included in, or close to, many real-life situations can contribute to a better transfer. Participation of proxies also ensures the use of acquired skills in everyday settings. If not task-oriented [4], therapy should at least aim at the implementation of the specific cognitive process in a given task, considering that the causal link between cognitive and linguistic processes is not clearly established yet. 

### 5.4. Partner-Based Action

A now acknowledged perspective focuses intervention efforts on communication partners. Behavioral intervention history has evolved in two models: “positive behavior supports model” ideally laud an intervention provided in natural community settings (home, work, and school). The primary providers should be the people who are natural communication partners in those settings (e.g., family members, work or school staff, peers), supported by specialists (therapists) focusing on lifestyle change satisfactory [66]. This collaborative work should start as soon as the assessment is initiated, and last throughout the therapy, to facilitate the generalization of acquired behaviors. 

### 5.5. Lexico-Semantics

Future studies could also focus on one of the unexplored components of language in terms of therapy, lexico-semantics, being at the origin of various difficulties observed at a higher language level. Joanette and colleagues [73] drew the initial conclusion that the right hemisphere could play an important role in lexical exploration strategies. According to Tompkins and Lehman [74], difficulties in interpreting words with ambiguous meaning and metaphorical expressions come from a deficit to inhibit the first activated meaning. In that perspective, RHD individual cannot detach from the primary, literal and concrete expressions to make an adequate interpretation. The alternative hypothesis, put forward by Beeman [75], is the coarse coding treatment of words by the right hemisphere. According to several authors, the right hemisphere is responsible for the activation of remote and less frequent semantic links [76, 77]. The supposed relations with mental flexibility are to be clarified.

### 5.6. Consideration for Specificities of the Aging Brain

After the age of 55, the risk of stroke doubles every 10 years [78] therefore, characteristics of the aging brain should be examined thoroughly. Park and Reuter-Lorenz [79] describe scaffolding as a normal process, acquired during learning. After a skilled performance is reached, the circuitry would shift to a more specific and optimal circuit of neural regions that are functionally interconnected. The initial scaffolding may then remain available as a secondary circuitry that can be recruited when performance takes place under challenge. With age, scaffolding, as a compensatory system, may be invoked to perform more familiar tasks and basic cognitive operations. The author mentions that “the maintenance of language in old age results from the continuous use of language and a particularly elaborate scaffolding network” [79, page 184].

It seems reasonable to infer that a right hemisphere damage could directly affect the compensatory circuitry (especially when occurring in the right DLPFC region frequently hosting scaffolding network), although especially necessary for compensation in critical time after stroke. That could partly explain why RHD might experience greater difficulties with high complexity material, which requires simultaneous integration of information, as it is the case in a conversation. In order to test this hypothesis, individual functional connectivity and pattern analysis should be run, considering that group analysis may be misleading when one is interested in patterns that are obviously very individualized [79].

In conclusion, treatments with RHD adults need to be structured according to actual theoretical and clinical knowledge in order to avoid any bias from attention and other cognitive disorders. Treatments should therefore include a hierarchy in terms of difficulty, use various modes of feedback, and take into account the concomitant cognitive disorders. Intervention should ideally be task-centered and as close as possible to real life, to reflect the complexity of communication in action and to allow generalization in many functional activities. Taking into consideration the poor knowledge regarding evidence-based treatment efficacy, studies with a stronger methodological design should be conducted by researchers with the collaboration of clinicians in order to specify the appropriate treatment methods for RHD individuals.
